# Supplementary material for: Content-rich biological network constructed by mining PubMed abstracts
Source: BMC Bioinformatics. 2004 Oct 8;5:147. doi: 10.1186/1471-2105-5-147 (PMC528731; doi:10.1186/1471-2105-5-147)
Supplement: Additional File 2 — The original results of the above study (non-essential files are deleted to keep the file size under the limit set by BMC bioinformatics). [file 1471-2105-5-147-S2.bz2 › chilibotAdditionalFile2/dip10/470ID9660793E10526/html/CCL7_CXCR3.html]

 


 **CCL7** and **CXCR3** 
  
Found 39 abstracts in PubMed, retrieved 10.  
 

 What does Google say? 
 PDF only 
| .edu only 

---

**Interactive relationship** (e.g. stimulation, inhibition, etc)

**Stimulatory relationship**- At 26 weeks postirradiation, messages encoding the chemokines BLC now known as Scyb13, C10 now known as Scya6  [ **CCL7** ] , IP 10  [ **CXCR3** ]  now known as Scyb10, MCP 1 now known as Scya2, MCP 3  [ **CCL7** ]  now known as Scya7  [ **CCL7** ] , MIP 1gamma now known as Scya9, and RANTES now known as Scya5 and the chemokine receptors Ccr1, Ccr2, Ccr5 and Ccr6 were elevated in fibrosis sensitive C57BL 6 mice.  Ref: 11839087 Radiat Res, 2002
- 1 LARC CCL20 whose mRNA level increased rapidly at 3 h post challenge and then gradually decreased, 2 JE CCL2, MARC  [ **CCL7** ]   **CCL7** , MIP 1gamma CCL9, monocyte chemoattractant protein MCP 5 CCL12, ELC CCL19 and BRAK CXCL14 whose mRNA levels increased with time and reached the maximum at 6 9 h post challenge, 3 LIX CXCL5, Mig CXCL9 and IP 10  [ **CXCR3** ]  CXCL10 whose mRNA levels increased gradually at least up to 12 h post challenge, and 4 SLC CCL21 whose mRNA level decreased gradually with time after challenge.  Ref: 12644322 Immunol Lett, 2003
**Neutral relationship**

**Non-interactive relationship** (e.g. studied together, co-existance, homology, etc.)

- Expression of IL 8, MCP 1, MCP 3  [ **CCL7** ] , and IP 10  [ **CXCR3** ]  was significantly higher in pouchitis than normal pouch.  Ref: 12827410 Int J Colorectal Dis< MedlineTA>Int J Colorectal Dis, 2003
- Because most of these genes have been known to either inhibit angiogenesis Mig, IP 10  [ **CXCR3** ] , inhibit tumor growth Gjb2, MCP3  [ **CCL7** ] , kill tumor cells Granzyme A and C, or induce expression of antitumor gene IRF 7, they may become promising therapeutic gene candidates for a combination gene therapy approach to cancer treatment.  Ref: 11960315 Gene Ther, 2002
- Analyses using cDNA array and Northern blot indicated that the genes responsible for the therapeutic effect of electro IFN alpha gene therapy included IRF 7, Granzyme A, Granzyme C, Gjb2, Krt14, Mig, IP 10  [ **CXCR3** ]  and MCP3  [ **CCL7** ] .  Ref: 11960315 Gene Ther, 2002
- Under unstimulated conditions, no differences in the production of interleukin 8 IL 8, IFN inducible protein 10 IP 10  [ **CXCR3** ] , monokine induced by interferon gamma Mig, monocyte chemoattractant protein 1 MCP 1, and monocyte chemoattractant protein 3  [ **CCL7** ]  MCP 3  [ **CCL7** ]  were seen between untreated MS patients and controls.  Ref: 12020972 J Neuroimmunol, 2002
- From among the 13 chemokines examined, MCP 3  [ **CCL7** ] , MCP 4, RANTES, and IP 10  [ **CXCR3** ]  were detected in the disc from the idiopathic scoliosis, and MCP 3  [ **CCL7** ] , MCP 4, RANTES, IP 10  [ **CXCR3** ] , MIG, and MGSA alpha were detected in the infected or herniated discs.  Ref: 12131709 Spine, 2002
- Biopsies were stained for CD68, CD3, elastase, eotaxin, IP 10  [ **CXCR3** ] , MCP 1, MCP 3  [ **CCL7** ] , and IL 8 and analyzed by NIH Image analyzer.  Ref: 12827410 Int J Colorectal Dis< MedlineTA>Int J Colorectal Dis, 2002
- Of the chemokines tested MIP 1alpha, MIP 1beta, MCP 1, MCP 3  [ **CCL7** ] , RANTES, SDF 1, IL 8, IP 10  [ **CXCR3** ] , only MIP 1alpha, detected specifically on microglia, was expressed both constitutively and consistently.  Ref: 11746784 Glia, 2002
- The expression of MCP 1, MCP 3  [ **CCL7** ]  and IP 10  [ **CXCR3** ]  were significantly lower after antibiotic treatment.  Ref: 12827410 Int J Colorectal Dis< MedlineTA>Int J Colorectal Dis, 2002
- The RNA was reverse transcribed, and the resultant cDNA was amplified by PCR using primer pairs specific to the CXC chemokines IL 8, MGSA alpha, IP 10  [ **CXCR3** ] , MIG, the CC chemokines MCP 1, MCP 2, MCP 3  [ **CCL7** ] , MCP 4, MIP 1alpha, MIP 3alpha, RANTES, STCP 1, the C chemokine lymphotactin, and the glyceraldehyde phosphate housekeeping gene.  Ref: 12131709 Spine, 2002
- In vitro addition of IFN beta1b to peripheral blood mononuclear cells PBMC cultures tended to decrease the production of IL 8, IP 10  [ **CXCR3** ] , Mig, MCP 1, and MCP 3  [ **CCL7** ] , but not of MIP 1alpha.  Ref: 12020972 J Neuroimmunol, 2002
